# Fatigue and Activity Management Education for Individuals with Systemic Lupus Erythematosus

**DOI:** 10.1155/2017/4530104

**Published:** 2017-01-11

**Authors:** Ruth O'Riordan, Michele Doran, Deirdre Connolly

**Affiliations:** ^1^Occupational Therapy Department, St. James' Hospital, James' Street, Dublin 8, Ireland; ^2^Rheumatology Department, St. James' Hospital, James' Street, Dublin 8, Ireland; ^3^Trinity Centre for Health Sciences, Discipline of Occupational Therapy, St. James' Hospital, James' Street, Dublin 8, Ireland

## Abstract

**Background:**

Fatigue and Activity Management Education (FAME) is a six-week occupational therapy-led programme focusing on fatigue and stress management, exercise, nutrition, and joint protection. Each session consists of education and goal setting.

**Objectives of Study:**

To assess the impact of FAME on occupational participation and fatigue management.

**Methods:**

Three programmes were facilitated with twenty-one women with SLE. A mixed methods design was used. Quantitative data were collected using self-reported questionnaires administered before, immediately after, and eight weeks after intervention. Data were analysed using descriptive and nonparametric inferential statistics. Qualitative data were collected through focus groups and interviews. Thematic analysis was carried out on the qualitative data.

**Findings:**

There was a statistically significant improvement in depression as measured by the Hospital Anxiety and Depression Scale and categories of “burden to others” and “fatigue” in the LupusQoL. There were nonsignificant improvements in fatigue, occupational participation, self-efficacy, and anxiety. Participants reported an improved understanding of fatigue and the impact of stress on fatigue. They also identified self-management strategies they were using on a daily basis.

## 1. Introduction

SLE is a complex autoimmune disease that can affect any organ in the body and display any array of clinical manifestations [1, 2]. Fatigue is one of the most prevalent symptoms of SLE affecting up to 90% of individuals even when the disease is in remission [3]. In chronic conditions such as SLE, fatigue is reported by individuals to be one of their most difficult symptoms and one that impacts the most on their quality of life [4, 5]. It is not known whether fatigue in SLE is a consequence of being chronically ill, or whether it represents a complication of the disease [6]. It is most common during periods of exacerbation and although it is assumed to reflect disease activity it also presents after the exacerbation has subsided, suggesting that other factors play a role [6].

Robinson Jr. et al. [7] identified that symptoms of SLE such as fatigue affect occupational engagement in household responsibilities, parenting roles, work performance, and scholastic achievement. Gallop et al. [8] reported that fatigue has a substantial impact on an individual's ability to perform self-care activities such as washing and dressing and has also has been shown to have a negative impact on participation in social and leisure activities. In a qualitative study on the impact of SLE-related fatigue on occupational participation, study participants reported no difficulties in self-care but identified a range of productivity and leisure activities which caused them difficulty [9].

Interventions for fatigue are generally categorised into pharmacological and nonpharmacological approaches. Drug therapies for SLE-related fatigue are a rapidly developing field with many recently completed or ongoing studies [10]. Although these studies are indicating promising results, potential side effects and high drug costs influence the decision to prescribe these medications.

Nonpharmacological interventions for fatigue are the most commonly used fatigue management strategies for chronic diseases [11]. These interventions are predominantly aimed at reducing the impact of pain and fatigue on occupational participation through development of effective self-management strategies [12]. Self-management is a dynamic process in which individuals actively manage their chronic illness [13]. It aims to maximise occupational performance and functioning by providing individuals with the skills to manage symptoms, treatments, and the psychological effects of living with a chronic disease such as SLE [14, 15].

Although a number of interventions are available for managing fatigue in chronic diseases, a recent study by Connolly et al. [9] reported that individuals with SLE identified a lack of input from health professionals on how to manage their fatigue. The study participants reported developing their fatigue management strategies through trial and error rather than focused education [9]. The authors concluded that early interventions on fatigue management strategies are warranted. Fatigue management programmes have been shown to impact positively on individuals with chronic diseases, yet there is a limited amount of studies documenting the benefit and effectiveness of fatigue self-management for individuals with SLE specifically [16].

A self-management programme, Fatigue and Activity Management Education (FAME), was therefore developed for people with SLE. Self-management programmes are recommended for people with SLE to develop cognitive, behavioural, and emotional strategies to achieve a satisfactory quality of life [17]. The overall aims of FAME are for participants to develop effective fatigue management strategies and increase their occupational participation.

A feasibility study, guided by the Medical Research Councils (MRC) Framework for complex interventions [18], was carried out to explore the impact of FAME on fatigue, occupational participation, mood, self-efficacy and quality of life. The study also explored participants' perceptions of the acceptability of the programme and its impact on management of their fatigue.

## 2. Methods

### 2.1. Intervention

FAME is facilitated by an occupational therapist with multidisciplinary input and consists of six (once per week) 2.5-hour sessions. Each session comprised one-hour group education and one-hour individual goal setting component with a 30-minute tea/coffee break between the two components. Educational topics included fatigue management, pain management, exercise (delivered by a physiotherapist), joint protection, stress management, and nutrition (delivered by a dietician). The goal setting component aimed to facilitate participants to implement learning gained during the educational session between the weekly sessions. The purpose of goal setting is to facilitate application of self-management skills to promote behaviour change [19].

A sequential explanatory mixed methods design was used for this study [20]. A quasi-experimental pretest and posttest design was conducted for the quantitative phase and a qualitative descriptive design for the qualitative phase.

### 2.2. Sampling

The inclusion criteria for this study were individuals with a definite diagnosis of SLE over the age of 18 as confirmed by a rheumatologist. Participants were recruited through a monthly lupus clinic in an urban teaching hospital through a chart review. Seventy individuals were identified as eligible and provided with an information leaflet. Of these, 59 expressed interest with 21 people participating in three separate FAME programmes delivered over a nine-month period. The three programmes consisted of four, five, and twelve participants per programme. Allocation to each programme was based on individuals' choice and their availability to attend on a particular day and time of the week. For the 38 people who expressed interest but did not attend, the main reasons given were unsuitable timing of the programme and geographical distance. Informed consent was obtained when participants attended preprogramme baseline assessments.

### 2.3. Data Collection Measures

The Fatigue Severity Scale (FSS) [21] measures severity of fatigue in individuals with SLE and Multiple Sclerosis (MS). The scale consists of nine questions and is short to administer. Responses to the nine statements are graded on a seven-point Likert scale with a score of seven indicating strong agreement and one indicating strong disagreement [22]. Scores of four or more indicate severe fatigue [23]. In patients with SLE the reliability of the FSS is acceptable for internal consistency and it is the recommended fatigue scale for SLE [24]. The FSS has strong internal consistency, reliability, and construct and criterion validity and is sensitive to change [21, 25].

The Energy Conservation Strategies Survey (ECSS) [26] is a 14-item self-administered instrument that measures implementation of energy conservation strategies. It is administered eight weeks after participation in fatigue management programmes to ascertain if participants are still engaging in strategies acquired during the programme [26]. Participants identify if they use specific strategies, the frequency of use, and perceived effectiveness on a scale of 1 (not effective) to 10 (very effective). Findings on the psychometric properties of the ECSS suggest that it exhibits high internal consistency and good test-retest reliability [26].

In the Self-Efficacy for Performing Energy Conservation Strategies Assessment (SEPECSA) [27] participants rate their level of confidence (from 1 to 10) in their ability to use energy conservation strategies with higher scores indicating greater confidence. The scale has documented reliability, validity and high internal consistency [28]. The SEPECSA was used in this study as self-efficacy is an integral component of self-management [29].

The Frenchay Activities Index (FIA) [30] is a behavioural scale that measures frequency of participation in social and instrumental activities of daily living. The FAI is divided into three subscales: domestic, leisure/work, and outdoor activities. The maximum score in each subscale is 15 with a total score of 15 to 45. Higher scores indicate greater frequency of performance in each area of activity [30]. The FAI was originally designed for use with individuals who experienced a stroke but has also been shown to be a valid and reliable instrument for well elderly populations and younger physically disabled populations [31]. Turnbull et al. [32] assessed the validity and reliability of the FAI in people aged 16 years and older and it was concluded that the FAI has good construct validity, particularly in middle aged and elderly people, and is reliable.

The Hospital Anxiety and Depression Scale (HADS) [33] is a self-report measure with an anxiety subscale and a depression subscale both containing seven items. Each item is rated on a 4-point scale from 0 (no problem) to 3 (severe problem). The maximum score is 21 for both anxiety and depression. Scores of 11 or more on either subscale indicate significant psychological morbidity. Scores of 8–10 represent borderline morbidity and scores of 0–7 indicate normal levels of anxiety and depression [33]. The HADS was originally developed for use in nonpsychiatric outpatient settings but it is also valid and reliable in the general population [34]. Studies have also confirmed the validity and reliability of the HADS for people with SLE [35, 36]. The HADS was chosen as a measure for this study as individuals with SLE are at risk of feelings of depression and anxiety related to increased disease activity and fatigue [6].

The Lupus Quality of Life Questionnaire (LupusQoL) contains 34 items across 8 subscales that are scored separately. It was developed and validated by using a mixed qualitative and quantitative approach [37]. Scores range from 0 (worst) to 100 (best). Of the available instruments to assess health related quality of life in individuals with SLE, the LupusQoL has undergone the most extensive validation process [38].

The Health Education Impact Questionnaire (HEIQ) [39] has 40 questions reflecting the aims of self-management, grouped into eight subscales, each scored separately [40]. Higher scores indicate stronger agreement. The HEIQ is a generic patient reported outcome measure developed as a psychometrically sound instrument for the evaluation of self-management programmes [40]. The development of the HEIQ involved extensive stakeholder consultation and rigorous statistical analysis [41].

Data collection occurred before intervention: Time 1 (*T*1); immediately after intervention: Time 2 (*T*2); and eight weeks after completion of FAME: Time 3 (*T*3). Descriptive and inferential statistics (nonparametric) were used to analyse the data at all three time points [20]. Quantitative data was analysed using Statistical Package for the Social Sciences [42]. Wilcoxon Signed Rank tests were measured for significant differences between each time point for each outcome. Twenty-one participants provided data at* T*1 and* T*2. However, six participants were lost to follow-up at* T*3 giving 15 people included in* T*3 data analysis.

### 2.4. Qualitative Data Collection

Focus groups and individual interviews were used to gather qualitative data. On the final session of each of the three FAME programmes a focus group explored acceptability of the content and delivery of FAME as per the MRC guidelines [18] and explored perceived impact of FAME on participants' fatigue management. All those present at the final session of each FAME programme were invited to participate in the focus groups. In total 19 individuals participated in the focus group discussions. All participants were also invited to participate in an interview eight weeks after completion of the programme (*T*3). However, due to work and family commitments, only six people were available to participate in the follow-up interviews. Open ended questions were developed based on data analysis of the quantitative measures and focus group data [43]. The following list contains sample questions for the focus groups and individual interviews.

*Focus Group and Interview Guide*

*Focus Group Questions*

  What was your overall impression of the programme?
  Which aspects of the programme did you find most useful and why?
  Which aspects of the programme did you find least useful and why?
  Has the programme helped you to understand your fatigue better?
    If so, in what way?
    If not, why not?
  Has the programme helped you to manage your fatigue better?
    If so, in what way?
    If not, why not?
  Have you any recommendations for improving the programme for future delivery?

*Interview Questions*

  How are you managing your fatigue now?
  Are you using any specific strategies from the programme to help you manage your fatigue?
    If so, which strategies are you using and how effective are they for you?
    If not, why not?
  Have you any recommendations for improving the programme for future delivery?

Focus groups and individual interviews were tape recorded to ensure verification of participant dialogue [44].

Analysis of qualitative data was completed using methods advised for qualitative description (QD). In QD, the aim of data analysis is to provide a rich, straight description of an experience or an event [45]. All qualitative data were transcribed and coded using NVIVO 10 QSR [46] package. To maximise rigour, two of the authors coded the transcripts separately and then came together to compare codes across the interviews and focus groups. Differences in codes were discussed and when there was disagreement, codes were agreed on and renamed and/or new codes were developed and applied across all transcripts. On completion of the coding process, codes were grouped into themes based on the study aims [43]. Copies of transcripts and a summary of the analysis were made available to participants to review to ensure that the interpretation of the data was consistent with experiences of the participants [47]. No changes were made by participants to their transcripts or the analysis summaries.

### 2.5. Ethics

Ethical approval was granted from the Ethics Committee of the hospital through which study participants were recruited.

## 3. Results

The mean age of participants was 48.1 years (Table 1). The mean number of years since diagnosis was 10.8 years. All participants were female and the majority were married (*n* = 10), living with others (*n* = 18), and employed (*n* = 12). The median number of FAME sessions attended was five.

### 3.1. Quantitative Results

Although the median FSS scores reduced slightly from* T*1 to* T*2 and remained the same at* T*3, there were no significant differences between the three time points. The median score of the FSS for all three time points was above four which signifies severe fatigue [21]. However, the proportion of participants who rated their fatigue as severe reduced from 90.5% (*n* = 19/21) at* T*1 to 80% (*n* = 12/15) at* T*3.

The majority of participants (*n* = 19) reported using all 14 energy conservation strategies listed in the ECSS at* T*3. The most commonly used strategies were “balancing rest and work,” “taking rest breaks,” and “resting during fatiguing activities”. The median effectiveness score for each of these strategies was 8/10. The most common strategies not used by participants were “changing work heights” and “changing location of items.” The main reason participants gave for not incorporating these strategies was that they were already implementing these prior to attending FAME.

The median SEPECSA score improved from* T*1 to* T*3 and, although not significant, the change was nearing significance (*p* = 0.056) indicating increased self-efficacy in using energy conservation strategies (Table 2). However, baseline scores were high indicating high self-efficacy prior to intervention which may have influenced a lack of significant changes across the three data points.

The FAI measures frequency of participation in self-care, productivity, and leisure. There were no statistically significant changes in FAI scores in any of the subscales or total score at any time point (Table 2). The baseline mean score of 31 out of a maximum of 45 indicated quite high levels of occupational participation for study participants. However, although the mean score increased at* T*2, it reduced below the* T*1 score at the eight-week follow-up indicating reduced participation in occupations eight weeks after completion of FAME.

The depression subscale of the HADS reduced over the period of the study with a significant change from* T*1 to* T*3 (*p* = 0.050). This change in mood is supported by the increase in the proportion of participants who scored within normal depression limits from 57% at* T*1 to 85% at* T*2. This increased further to 93% at* T*3. Although no significant changes were found in the anxiety scale, 14% of participants had severe anxiety at* T*1 and this reduced to 0% by* T*2 and remained at this at* T*3.

In the Lupus Quality of Life Scale (LupusQoL), all categories improved between* T*1 and* T*2 except “body image.” Three categories, namely, “physical health,” “burden to others,” and “fatigue,” improved significantly between* T*1 and* T*2. However all categories (except “body image”) regressed to baseline between* T*2 and* T*3 with significant reductions in “physical health,” “burden to others,” and “fatigue” (Table 3).

The majority of categories of the HEIQ improved between* T*1 and* T*2 with three categories, “positive and active engagement in life,” “skill and technique acquisition,” and “self-monitoring and insight,” improving significantly. The category of “skill and technique acquisition” remained significant between* T*1 and* T*3 (Table 4).

### 3.2. Qualitative Results

Three themes were identified following analysis of the qualitative data:

1. Validation of fatigue
2. Peer support
3. Application of learning

#### 3.2.1. Validation of Fatigue

People with fatigue often express a lack of understanding from family, friends, and work colleagues of their fatigue. During the focus groups, some participants described a similar lack of understanding from others of their fatigue:

P17:* The tiredness is terrible sometimes and I suppose nobody understands why, because I look great. Sometimes I look great, like I'm full of energy, but I'm not*. (interview)

P3:* I've quite a wide circle of friends and they all know there's something wrong with me, but they don't really understand what it is. I'm afraid people think I'm lazy. You don't like to feel different. I don't want people thinking there's something wrong with me.* (focus group)

Through attending FAME and meeting others with SLE, participants reported validation of their fatigue through others in the group reporting similar levels of fatigue.

P2:* Finally somebody's exactly the same as me. It's nice to see you're not on your own*. (focus group)

P5:* Realising that other people have this, and sharing that, was great*. (interview)

People with lupus have reported the importance of changing their attitude to fatigue and learning to accept this symptom as an important element of managing their fatigue [9]. Participants in this study described how attending FAME helped them to accept their fatigue:

P1:* After going to the group I felt it was much easier to accept it because of listening to all the others. *(interview)

One participant described the emotions she experienced in relation to her fatigue and how it prevented her from using fatigue management strategies and affected her engagement in activities.

P13:* I used to feel guilty if I couldn't do something, really guilty, and I wouldn't lie down. But now I know that everybody is similar and the one thing that everybody has in common is fatigue. *(focus group).

#### 3.2.2. Peer Support

Peer support has been identified as an important factor in facilitating health behaviour changes in health education programmes [48]. Participants discussed the benefit of learning from each other. They described comparisons and differences between each other and reported that these observations helped to influence behaviour change.

P12:* I've learned a lot from everyone. Everyone had different levels of fatigue. Some people are worse than you and some are better. Everyone has good and bad days* (focus group).

P5:* Realising that other people have this and sharing that was great*. (interview)

P1:* Listening to others that had the same problem was good, I wasn't on my own*. (interview)

Group education programmes are also an opportunity for participants to compare their level of functioning with others. For example, one participant perceived herself as having less severe disease than others in her group:

P8:* It has helped me realise just how lucky I am compared to other participants*. (focus group)

P16:* I think as well hearing everyone else's story, the support as a group has been really beneficial, and just for me, realising that I'm not superwoman*. (focus group)

#### 3.2.3. Application of Learning

Participants described how they applied learning acquired from FAME to their daily routine and the positive effect this had on managing occupational participation. Some participants reported using fatigue management strategies such as pacing and prioritising:

P1:* I'm trying to do a few things that we talked about, for example to do things and then sit down and have a rest before you have to do something else*. (interview)

P9:* I picked up on what was said about instead of trying to do a load of work all together and then knocking yourself for six, just make a plan, how much can I do today and leave the rest until tomorrow.* (focus group)

Taking frequent rest breaks was a fatigue management strategy recommended to FAME participants. One participant described how she incorporates this strategy into her daily activities:

P17:* I was inclined to keep on doing things until I was really too tired but I'd want to get it finished. But now I know that I have to stop and take time out for myself*. (focus group)

In her follow-up interview, Participant 17 discussed using the strategy of taking rest breaks.

P17: Now I just say* “I'm going up to lie down” and I don't care what anyone thinks*. (interview)

Some participants described incorporating joint protection principles when preparing meals. For example,

P14:* A few little tips I didn't even think of like pulling or sliding the kettle across the counter instead of lifting it.* (focus group)

Using labour saving devices can also reduce fatigue; however some participants reported not using equipment that had been provided to them. However, one participant described that since attending FAME she has a different attitude to using her stair lift:

P12:* I have a stair-lift, which I always refused to use, because to me if I'm not able to go up the stairs, well, I'd nearly crawl up quicker than use it. Whereas lately if I feel if I can't get up the stairs, I am using it. It's not an admission of “I can't do this”, it's, “today I can't, but tomorrow I will.”* (focus group)

Delegation is another energy conservation strategy discussed during FAME. Some participants described the benefits of using this strategy. For example,

P19:* You are allowed to delegate and you feel better for it. Definitely! So, I'll keep doing it now.* (focus group)

P2:* It's the way I'm doing things, because now I'm delegating, “Take the washing out of the washing machine for me please*.” (interview)

Another element of the programme that participants discussed as helpful was stress management. The content of one of the six sessions of FAME focuses on causes of stress and anxiety and practices relaxation strategies that participants can use at home or in work. In the focus group some participants identified this as beneficial. For example, one participant described increased awareness of managing stress:

P16:* It has helped me to reach an understanding that I must start looking after myself and also to find ways to relax and unwind and find some quiet time, some “me-time.”* (focus group)

In a follow-up interview, another participant identified how she recognised improvements in her mood when completing the follow-up HADS questionnaire:

P2:* I'm less anxious now. When I first filled it out (HADS) I was so depressed. Yes, there is much more of a change, I'm a bit more up-beat and a bit more positive.*

Overall participants of the qualitative phase of the study identified benefits of meeting other people with SLE and receiving validation that their fatigue is a recognised symptom of SLE. Participants described how fatigue and stress management strategies acquired during FAME were affecting their activity management positively.

## 4. Discussion

The aim of FAME (Fatigue and Activity Management Education) was to increase participants' understanding of SLE-related fatigue and to facilitate development of strategies to decrease the impact of fatigue on occupational participation. Three six-week FAME programmes were delivered to a total of 21 people over a 9-month period. Thirty-eight individuals who originally expressed interest during recruitment did not participate in FAME. Perhaps these participants did not feel ready to take part in self-management and may need support in making the transition from precontemplation to contemplation of adopting self-management strategies [17]. The FAME programme was offered to men and women; however only women participated. SLE is more common in women than men with a typical female to male ratio of 9 : 1. Despite the increased prevalence of SLE in women, offering male only groups, telephone programmes, or online programmes may facilitate increased male engagement [8]. Six participants were lost to the eight-week follow-up stage despite numerous attempts to contact them.

Fatigue was measured using the Fatigue Severity Scale (FSS) and is also included as a category in the LupusQoL scale. There were no statistically significant differences in the FSS scores from the beginning to the end of the study although the proportion of participants with severe fatigue (scores ≥ 4) decreased from 91% at the beginning of FAME to 80% at the eight-week follow-up stage. Although the fatigue category in the LupusQoL improved significantly between baseline and immediately after FAME, this was not sustained at eight-week after FAME.

A possible reason for the lack of significant changes in the FSS scores may be that as fatigue is a constant symptom for up to 90% of people with SLE, it may not be possible to eliminate this symptom through a self-management programme. Therefore perhaps the FSS was not the most suitable measure to use as it is a unidimensional scale which measures severity of fatigue rather than the impact of fatigue on participants' daily occupations. For this reason a more appropriate measure for evaluating fatigue management programmes would be a multidimensional measure such as the Fatigue Impact Scale [49]. Further research is required to confirm this possibility.

As stated earlier the aim of FAME is to increase participants' understanding of their fatigue rather than reducing its severity. One of the categories of the Health Education Impact Questionnaire (HEIQ) measures participants' ability to self-monitor their health condition and levels of insight. There were significant improvements in this category between the beginning and the end of FAME but this was not sustained at eight weeks. This suggests that perhaps a 6-week programme is not long enough to support sustained changes in fatigue management strategies or that participants may need individual and/or ongoing support after completion of FAME. However, in the follow-up qualitative phase of the study, participants reported that they had learned to accept their fatigue and not push themselves to complete activities when they experienced fatigue. It appears therefore that FAME may have achieved its goal of improved awareness and understanding of fatigue; however, further research is required to confirm this finding.

As previously outlined, FAME includes educational sessions related to the role of exercise, diet, and stress in fatigue management. These topics are included in FAME based on findings from a qualitative study with women with SLE who identified difficulties in these three areas [9]. Research evidence also supports the impact of these lifestyle factors on effectively managing fatigue [10]. The impact of including these topics on fatigue levels was not specifically measured in this study; however, future research could measure the impact of these three elements on fatigue using either qualitative or quantitative research methods.

Another possible explanation for the small reduction in FSS scores is that perhaps participants had underrated their fatigue at the baseline assessment period but by the end of FAME, through their increased awareness and understanding of fatigue, they provided a more candid and accurate rating of their fatigue. This was a similar finding to a fatigue self-management programme for people with Multiple Sclerosis [50]. This finding could also indicate that perhaps using an outcome of fatigue severity is not appropriate for self-management of SLE-related fatigue and that outcome measures need to capture changes in attitude towards fatigue and measure levels of insight of the impact of fatigue on occupational performance. The Canadian Measure of Occupational Performance [51], which measures levels of satisfaction with occupational performance, may be a suitable measure to capture these outcomes.

There was a statistically significant reduction in the depression category of the HADS measure between the beginning of FAME and eight weeks after FAME. This improvement in mood was also indicated through a significant improvement in participants' scoring in the category of “burden to others” in the Lupus Quality of Life Scale between the beginning and end of FAME. This category explores the extent to which participants believe they cause stress to others around them and are a burden to their family. In the qualitative phase of the study, participants discussed how attending FAME, and listening to others discussing their fatigue, validated for them that fatigue is a recognised symptom of SLE. This validation process may have been a contributing factor to improved mood as many participants reported a lack of understanding from others regarding the authenticity of their fatigue. Participants also discussed using stress management strategies acquired during FAME to manage their stress and anxiety. Research in SLE has reported a relationship between fatigue and depression but has not as yet established if one precedes the other [52]. However, as this was a feasibility study, further research using a control group is required to examine a cause and effect between attendance at FAME and changes in mood.

The HEIQ examines participants' confidence in applying skills attained through self-management education [39]. In this study, the category of “skill and technique acquisition” improved significantly from baseline to immediately after FAME and this significant improvement was maintained from baseline to the eight-week follow-up. In their follow-up interviews participants were asked which, if any, energy management strategies acquired through FAME they were currently using. Interviewees identified a range of strategies covered in FAME including pacing activities, using joint protection principles such as avoiding lifting heavy objects and using labour saving equipment. Each FAME session included a goal setting component where each participant was required to set a goal related to the weekly FAME content in order to practice skills related to fatigue management. Goal setting is a recognised method of facilitating health behaviour changes [53]. Through the weekly goal setting process participants were encouraged to try the energy management strategies at home or in work environments and to give feedback to the facilitators the following week on how effective the strategies were for them. Perhaps through actively practising the various fatigue management strategies participants were facilitated to apply these strategies over longer periods of time.

There were no significant improvements in occupational participation over the three time points where FAI scores either remained the same or reduced slightly. This reduction in occupational participation could be viewed as a negative outcome of FAME given the importance of occupational participation for physical and psychological wellbeing [51]. However, this reduction may also be related to participants making different choices about the occupations in which they choose to engage. Previous research reported that people with SLE made decisions to reduce their participation in certain occupations in order to have energy to engage in what they considered more important occupations [9]. This possibility needs further research to confirm this explanation.

The findings from the ECSS indicated that the top three energy conservation strategies for FAME participants related to resting more frequently and taking short rest breaks during fatiguing activities. This energy management strategy is a frequent recommendation for those experiencing fatigue [11]. If this is the case, perhaps this reduced the time that participants had over the course of their day to engage in the same number of occupations as prior to attending FAME. Delegation of physically demanding occupations is also a frequent recommendation for energy management which could also explain participants' reduced occupational participation levels. This reduction could therefore be considered a positive outcome as, following FAME, perhaps participants stopped pushing themselves to perform activities that increased their fatigue.

Self-efficacy is defined as an individual's belief in their ability to manage their health condition and having the necessary skills and knowledge to manage the impact of their health on their daily lives [53]. By providing people with knowledge and skills to manage SLE-related fatigue, FAME aims to increase participants' self-efficacy. Although approaching significance at the eight-week follow-up assessment, there were no significant changes in self-efficacy between the three data collection points. However, development of positive self-efficacy is dependent on repeated experiences of successes over time and perhaps an eight-week follow-up is not sufficient time to capture the impact of FAME on self-efficacy. Perhaps individual follow-up sessions with participants may facilitate increased confidence in using energy management strategies. However, other self-management programmes have reported similar findings indicating that a three- or six-month follow-up period may be required to capture significant changes in self-efficacy [54].

## 5. Conclusion and Recommendations for Future Research

A six-week fatigue management programme, FAME, was designed to improve participants' understanding of fatigue and to provide strategies to manage SLE-related fatigue. Although there were improvements in fatigue severity, these were not significant. However, participants' reported that their awareness and attitude to fatigue improved and discussed applying energy management strategies to occupational participation up to eight weeks following FAME. Given the enduring nature of SLE-related fatigue, the ability to self-monitor levels of fatigue and possess a range of energy management skills is an important self-management strategy for long term management of SLE-related fatigue.

There was a significant improvement in participants' depression levels from the beginning of FAME to the eight-week follow-up period. There are a number of potential contributing factors for this improvement such as validation of fatigue as a problematic symptom of SLE and acquisition of effective fatigue and stress management strategies through participation in FAME. Mental health difficulties are reported in up to 51% of people with SLE [55]. This indicates the need for interventions to relieve symptoms, such as fatigue, that are associated with reduced mood.

One of the aims of FAME is to increase occupational participation; however this reduced from the end of FAME to the eight-week follow-up. Rather than considering this as a negative outcome, perhaps participants were conserving energy by withdrawing their participation in certain occupations in order to have adequate energy to engage in more valued occupations. However, this finding could also be related to participants requiring a longer programme duration than that provided by FAME. Further research is required to examine these possibilities.

Based on the findings of this study, a larger trial is the next recommended stage in the MRC framework for developing complex interventions [18]. However, despite the lack of a control group and the small sample size, this study indicates that FAME is a promising intervention for people with SLE to develop knowledge and skills for self-management of fatigue.

## Figures and Tables

**Table 1:** Participant demographics.

Demographics	*n* = 21
Age	
(i) Mean	48.05 years
(ii) Standard deviation	15.25 years
(iii) Range	26–88 years

Length of time since diagnosis of lupus	
(i) Mean	10.8 years
(ii) Standard deviation	10.7 years
(iii) Median	8.0 years
(iv) Range	1–45 years

Relationship status	
(i) Single	8 (38.1%)
(ii) Married	10 (47.6%)
(iii) Widowed	1 (4.8%)
(iv) Divorced	1 (4.8%)
(v) Separated	1 (4.8)

Living situation	
(i) Alone	3 (14.3%)
(ii) With others	18 (85.7%)

Children	
(i) Yes	9 (42.9%)
(ii) No	12 (57.1%)

Employment status	
(i) Employed	12 (57.1%)
(ii) Self-employed	9 (42.9%)
(iii) Unemployed	

Number of sessions attended	
(i) Median	5
(ii) Range	3–6

**Table 2:** Median scores FSS, FAI, HADS, and SEPECSA at *T*1, *T*2, and *T*3.

Outcome measure	*T*1 Median (Range) *n* = 21	*T*2 Median (Range) *n* = 21	*T*3 Median (Range) *n* = 15	*T*1/*T*2 *p* value	*T*2/*T*3 *p* value	*T*1/*T*3 *p* value
FSS	5.33 (1.9–6.9)	5.11 (3.4–6.4)	5.11 (2.9–6.2)	0.370	1.000	0.306

FAI	31 (22–41)	32 (16–41)	29 (14–39)	0.726	0.609	0.753

SEPECSA	7.5 (2.6–10)	7.42 (3.4–10)	7.78 (4.3–10)	0.126	0.4572	0.056

HADS-A	8 (4–20)	10 (3–14)	7 (2–18)	0.722	0.229	0.342

HADS-D	6 (1–13)	5 (1–12)	4 (2–14)	0.880	0.823	*0.050*

**Table 3:** LupusQol *T*1, *T*2, and *T*3 changes.

Subscales	*T*1 median (Range) *n* = 21	*T*2 median (Range) *n* = 21	*T*3 median (Range) *n* = 15	*T*1/*T*2 *p* value	*T*2/*T*3 *p* value	*T*1/*T*3 *p* value
Physical health	57.14 (18.7–96.7)	61.46 (18.7–84.4)	52.5 (28.1–84.4)	*0.042*	*0.044*	0.875

Pain	61.90 (0–100)	69.84 (8.3–100)	58.89 (8.3–100)	0.107	0.624	0.208

Planning	61.51 (6–100)	64.29 (16.5–91.5)	56.67 (0–100)	0.959	0.900	0.550

Intimate relationships	69.64 (0–100)	75.00 (0–100)	60.83 (8.3–100)	0.244	0.062	0.107

Burden to others	53.17 (0–100)	63.10 (0–100)	55 (42.5–100)	*0.046*	*0.033*	0.195

Emotional health	72.22 (25–100)	77.18 (37.5–100)	74.72 (29.3–100)	0.199	0.778	0.507

Body image	75.48 (25–100)	74.76 (25–100)	75.33 (20–100)	0.835	0.387	0.875

Fatigue	38.99 (0–75)	44.94 (12.5–81.3)	34.58 (6.3–81.3)	*0.016*	*0.044*	0.860

**Table 4:** HEIQ *T*1, *T*2, and *T*3 changes.

HEIQ	*T*1 Median (Range) *n* = 21	*T*2 Median (Range) *n* = 21	*T*3 Median (Range) *n* = 15	*T*1/*T*2 *p* value	*T*2/*T*3 *p* value	*T*1/*T*3 *p* value
Positive and active engagement in life	13 (9–20)	15 (12–19)	15 (10–20)	*0.030*	0.621	0.123

Health directed behaviour	11 (7–15)	12 (9–16)	12 (8–16)	0.065	0.765	0.151

Skill and technique acquisition	11 (6–16)	12 (11–15)	12 (10–16)	*0.003*	1.000	*0.018*

Constructive attitudes and approaches	15 (10–20)	15 (13–20)	15 (14–20)	0.212	0.811	0.098

Self-monitoring and insight	18 (12–23)	19 (18–24)	19 (17–24)	*0.014*	0.905	0.106

Health service navigation	15 (10–20)	15 (5–19)	15 (8–20)	0.917	0.608	0.788

Social interaction and support	14 (9–19)	15 (11–19)	15 (10–20)	0.057	0.745	0.182

Emotional wellbeing	13.5 (8–23)	14 (8–19)	13 (7–19)	0.121	0.778	0.394

## Additional Points

*Relevance to Clinical Practice*. An occupational therapy-led self-management programme can facilitate improved mood and application of fatigue management strategies to occupational performance.* Limitations and Recommendations for Further Research. *The small sample size and lack of randomisation reduces the rigour of this study. Future research should incorporate a randomised controlled trial.

## References

[1] O'Neill S., Cervera R. (2010). Systemic lupus erythematosus. *Best Practice and Research: Clinical Rheumatology*.

[2] Pons-Estel G. J., Alarcón G. S., Scofield L., Reinlib L., Cooper G. S. (2010). Understanding the epidemiology and progression of systemic lupus erythematosus. *Seminars in Arthritis and Rheumatism*.

[3] Cleanthous S., Tyagi M., Isenberg D. A., Newman S. P. (2012). What do we know about self-reported fatigue in systemic lupus erythematosus?. *Lupus*.

[4] Connolly D., O'Toole L., Redmond P., Smith S. M. (2013). Managing fatigue in patients with chronic conditions in primary care. *Family Practice*.

[5] Finlayson M., Preissner K., Cho C. (2012). Outcome moderators of a fatigue management program for people with multiple sclerosis. *American Journal of Occupational Therapy*.

[6] Moldovan I., Cooray D., Carr F. (2013). Pain and depression predict self-reported fatigue/energy in lupus. *Lupus*.

[7] Robinson D., Aguilar D., Schoenwetter M. (2010). Impact of systemic lupus erythematosus on health, family, and work: the patient perspective. *Arthritis Care & Research*.

[8] Gallop K., Nixon A., Swinburn P., Sterling K. L., Naegeli A. N., Silk M. (2012). Development of a conceptual model of health-related quality of life for systemic lupus erythematosus from the patient's perspective. *Lupus*.

[9] Connolly D., McNally A., Moran D., Ryan M. (2014). Fatigue in systemic lupus erythematosus: impact on occupational participation and reported management strategies. *British Journal of Occupational Therapy*.

[10] Yuen H. K., Cunningham M. A. (2014). Optimal management of fatigue in patients with systemic lupus erythematosus: a systematic review. *Therapeutics and Clinical Risk Management*.

[11] Harrison S. (2007). *Fatigue Management for People with Multiple Sclerosis*.

[12] Del Pino-Sedeño T., Trujillo-Martín M. M., Ruiz-Irastorza G., Cuellar-Pompa L., De Pascual-Medina A. M., Serrano-Aguilar P. (2016). Effectiveness of nonpharmacologic interventions for decreasing fatigue in adults with systemic lupus erythematosus: a systematic review. *Arthritis Care and Research*.

[13] Schulman-Green D., Jaser S., Martin F. (2012). Processes of self-management in chronic illness. *Journal of Nursing Scholarship*.

[14] Bourbeau J. (2008). Clinical decision processes and patient engagement in self-management. *Disease Management and Health Outcomes*.

[15] Lorig K. R., Sobel D. S., Ritter P. L., Laurent D., Hobbs M. (2001). Effect of a self-management program on patients with chronic disease. *Effective Clinical Practice*.

[16] Neill J., Belan I., Ried K. (2006). Effectiveness of non-pharmacological interventions for fatigue in adults with multiple sclerosis, rheumatoid arthritis, or systemic lupus erythematosus: a systematic review. *Journal of Advanced Nursing*.

[17] Barlow J., Wright C., Sheasby J., Turner A., Hainsworth J. (2002). Self-management approaches for people with chronic conditions: a review. *Patient Education and Counseling*.

[18] Craig P., Dieppe P., Macintyre S., Mitchie S., Nazareth I., Petticrew M. (2008). Developing and evaluating complex interventions: the new Medical Research Council guidance. *British Medical Journal*.

[19] Park M. J., Green J., Ishikawa H., Kiuchi T. (2013). Hidden decay of impact after education for self-management of chronic illnesses: hypotheses. *Chronic Illness*.

[20] Creswell J. W. (2009). *Research Design: Qualitative, Quantitative, and Mixed Methods Approaches*.

[21] Krupp L. B., LaRocca N. G., Muir-Nash J., Steinberg A. D. (1989). The fatigue severity scale. Application to patients with multiple sclerosis and systemic lupus erythematosus. *Archives of Neurology*.

[22] Mattsson M., Möller B., Lundberg I. E., Gard G., Boström C. (2008). Reliability and validity of the fatigue severity scale in Swedish for patients with systemic lupus erythematosus. *Scandinavian Journal of Rheumatology*.

[23] Anton H. A., Miller W. C., Townson A. F. (2008). Measuring fatigue in persons with spinal cord injury. *Archives of Physical Medicine and Rehabilitation*.

[24] Hewlett S., Dures E., Almeida C. (2011). Measures of fatigue: Bristol Rheumatoid Arthritis Fatigue Multi-Dimensional Questionnaire (BRAF MDQ), Bristol Rheumatoid Arthritis Fatigue Numerical Rating Scales (BRAF NRS) for Severity, Effect, and Coping, Chalder Fatigue Questionnaire (CFQ), Checklist Individual Strength (CIS20R and CIS8R), Fatigue Severity Scale (FSS), Functional Assessment Chronic Illness Therapy (Fatigue) (FACIT-F), Multi-Dimensional Assessment of Fatigue (MAF), Multi-Dimensional Fatigue Inventory (MFI), Pediatric Quality Of Life (PedsQL) Multi-Dimensional Fatigue Scale, Profile of Fatigue (ProF), Short Form 36 Vitality Subscale (SF-36 VT), and Visual Analog Scales (VAS). *Arthritis Care and Research*.

[25] Dittner A. J., Wessely S. C., Brown R. G. (2004). The assessment of fatigue: a practical guide for clinicians and researchers. *Journal of Psychosomatic Research*.

[26] Mallik P. S., Finlayson M., Mathiowetz V., Fogg L. (2005). Psychometric evaluation of the energy conservation strategies survey. *Clinical Rehabilitation*.

[27] Liepold A., Mathiowetz V. (2005). Reliability and validity of the self-efficacy for performing energy conservation strategies assessment for persons with multiple sclerosis. *Occupational Therapy International*.

[28] Swain M. G. (2000). Fatigue in chronic disease. *Clinical Science*.

[29] Lorig K., Holman H., Sobel D., Laurent D., Gonzales V., Minor M. (2006). *Living a Healthy Life with Chronic Conditions*.

[30] Holbrook M., Skilbeck C. E. (1983). An activities index for use with stroke patients. *Age and Ageing*.

[31] Wu C.-Y., Chuang L.-L., Lin K.-C., Horng Y.-S. (2011). Responsiveness and validity of two outcome measures of instrumental activities of daily living in stroke survivors receiving rehabilitative therapies. *Clinical Rehabilitation*.

[32] Turnbull J. C., Kersten P., Habib M., McLellan L., Mullee M. A., George S. (2000). Validation of the Frenchay Activities Index in a general population aged 16 years and older. *Archives of Physical Medicine and Rehabilitation*.

[33] Zigmond A. S., Snaith R. P. (1983). The hospital anxiety and depression scale. *Acta Psychiatrica Scandinavica*.

[34] Gale C. R., Allerhand M., Sayer A. A. (2010). The structure of the hospital anxiety and depression scale in four cohorts of community-based, healthy older people: the HALCyon program. *International Psychogeriatrics*.

[35] Snaith R. P. (2003). The hospital anxiety and depression scale. *Health Quality of Life Outcomes*.

[36] Smarr K. L., Keefer A. L. (2011). Measures of depression and depressive symptoms: beck depression inventory-II (BDI-II), center for epidemiologic studies depression scale (CES-D), geriatric depression scale (GDS), hospital anxiety and depression scale (HADS), and patient health questionnaire-9 (PHQ-9). *Arthritis Care and Research*.

[37] McElhone K., Abbott J., Shelmerdine J. (2007). Development and validation of a disease-specific health-related quality of life measure, the LupusQoL, for adults with systemic lupus erythematosus. *Arthritis Care & Research*.

[38] Yazdany J. (2011). Health-related quality of life measurement in adult systemic lupus erythematosus: Lupus Quality of Life (LupusQoL), Systemic Lupus Erythematosus-Specific Quality of Life Questionnaire (SLEQOL), and Systemic Lupus Erythematosus Quality of Life Questionnaire (L-QoL). *Arthritis Care and Research*.

[39] Osborne R. H., Elsworth G. R., Whitfield K. (2007). The Health Education Impact Questionnaire (heiQ): an outcomes and evaluation measure for patient education and self-management interventions for people with chronic conditions. *Patient Education and Counseling*.

[40] Schwarze M., Kirchhof R., Schuler M., Musekamp G., Ehlebracht-Konig I., Gutenbrunner C. (2009). Evaluation of the impact of patient education for people with chronic conditions: translation and adaptation of the health education impact questionnaire (HEIQ) in Germany. *International Journal of Rehabilitation Research*.

[41] Morita R., Arakida M., Osborne R. H., Nolte S., Elsworth G. R., Mikami H. (2013). Adaptation and validation of the Japanese version of the Health Education Impact Questionnaire (heiQ-J) for the evaluation of self-management education interventions. *Japan Journal of Nursing Science*.

[42] IBM Corporation (2011). *IBM SPSS Statistics for Windows, Version 20.0*.

[43] Liamputtong P. (2009). *Qualitative Research Methods*.

[44] Shenton A. K. (2004). Strategies for ensuring trustworthiness in qualitative research projects. *Education for Information*.

[45] Neergaard M. A., Olesen F., Andersen R. S., Sondergaard J. (2009). Qualitative description—the poor cousin of health research?. *BMC Medical Research Methodology*.

[46] (2012). *NVivo Qualitative Data Analysis Software, Version 10*.

[47] Curtin M., Fossey E. (2007). Appraising the trustworthiness of qualitative studies: guidelines for occupational therapists. *Australian Occupational Therapy Journal*.

[48] Rollnick S., Mason P., Butler C. (2000). *Health Behaviour Change: A Guide for Practitioners*.

[49] Fisk J. D., Ritvo P. G., Ross L., Haase D. A., Marrie T. J., Schlech W. F. (1994). Measuring the functional impact of fatigue: initial validation of the fatigue impact scale. *Clinical Infectious Diseases*.

[50] Stapleton T., Mulholland L. (2004). Assessment of a fatigue management programme for people with multiple sclerosis. *International Journal of Therapy and Rehabilitation*.

[51] Law M., Baptiste S., Carswell A., McColl M. A., Polatajko H., Pollock N. (1998). *Canadian Occupational Performance Measure (COPM)*.

[52] Beckerman N. L., Auerbach C., Blanco I. (2011). Psychosocial dimensions of SLE: implications for the health care team. *Journal of Multidisciplinary Healthcare*.

[53] Lorig K. R., Holman H. R. (2003). Self-management education: history, definition, outcomes, and mechanisms. *Annals of Behavioral Medicine*.

[54] O’Toole L., Connolly D., Smith S. (2013). Impact of an occupation-based self-management programme on chronic disease management. *Australian Occupational Therapy Journal*.

[55] Pettersson S., Boström C., Eriksson K., Svenungsson E., Gunnarsson I., Henriksson E. W. (2015). Lifestyle habits and fatigue among people with systemic lupus erythematosus and matched population controls. *Lupus*.

